# Unwelcome Guest: PBDEs in Indoor Dust

**DOI:** 10.1289/ehp.116-a202

**Published:** 2008-05

**Authors:** Kellyn S. Betts

Researchers have known for years that house dust is a major exposure route for lead and certain pesticides. Now attention is turning to another class of dustborne chemicals—polybrominated diphenyl ether (PBDE) flame retardants. A growing body of research documents that PBDEs and other brominated flame retardants (BFRs) released from many different consumer products can accumulate in people’s homes, cars, and workplaces. Moreover, certain segments of the population have extremely high concentrations of these substances in their bodies. However, hard data on the human health impact of these exposures are only just beginning to emerge, with many studies focusing on thyroid effects.

PBDEs have been used extensively in the highly flammable plastic components of consumer goods including couches, mattresses, carpet padding, televisions, computers, cushions, car stereos, navigation systems, car seats, and padded dashboards. By 2001, a sufficient volume of data documenting PBDEs’ persistence, toxicity, and tendency to bioaccumulate had emerged that Europeans were calling for two PBDE formulations—pentaBDE and octaBDE—to be banned. Both formulations are mixtures of individual PBDE compounds, or congeners; they are named on the basis of the average number of bromine atoms making up the majority of each mixture. PentaBDE was used in cushioning material whereas octaBDE was used primarily in electronics including televisions, computers, and cell phones.

Europe went on to ban both PBDEs in August 2004, and the bromine and flame retardant industries also voluntarily ceased production in North America by the end of that year. PentaBDE and octaBDE are now candidates for inclusion in the United Nation’s Stockholm Convention on Persistent Organic Pollutants (POPs), which globally bans chemical substances that bioaccumulate through the food web and pose a risk to humans and the environment.

A third PBDE, an additive known as decaBDE, is used in electronic devices and textile backing. It remains in use today in North America, but was banned on 1 April 2008 by the European Court of Justice. The Bromine Science and Environmental Forum (BSEF), an industry group, is contesting that ruling, and writes on its website: “After 10 years of scientific research and more than 588 studies conducted and/or reviewed, both the environment and human risk assessment reports concluded that there is no significant risk [for decaBDE].” However, animal research suggests the compound may be carcinogenic and links it with developmental toxicity.

## PBDEs in the U.S. Population

PBDEs differ from most other POPs in two important and interrelated ways. The first is source of exposure. Whereas food is the main source of most of the persistent pollutants that people take up—including polychlorinated biphenyls (PCBs), mercury, and pesticides such as DDT—study after study suggests that consumer products are the main sources of PBDEs that have been documented in indoor dust. For this reason, researchers have begun to call PBDEs “indoor POPs.” The major role played by household dust helps explain another aspect of these compounds that makes them unlike other POPs: their uneven distribution in human populations.

In the 15 February 2008 issue of *Environmental Science & Technology*, Andreas Sjödin, a chemist with the Centers for Disease Control and Prevention (CDC), and colleagues published data from the National Health and Nutrition Examination Survey (NHANES) depicting the first nationally representative analysis of U.S. citizens’ blood for PBDEs. The publication of these data, which are based on blood samples collected in 2003 and 2004, makes the United States the first country in the world to have nationally representative data on the flame retardants.

The new findings suggest that U.S. citizens harbor levels of PBDEs that are much higher—between 7.1 and 35 times, considering the median concentrations of BDE-47, the congener detected most frequently—than Europeans, based on smaller, nonrepresentative studies. BDE-47, which is associated with the pentaBDE formulation, was found in 98.8% of samples tested. The NHANES data document that the youngest Americans participating in the study (aged 12–19 years) tended to have the highest overall concentrations of PBDEs in their blood as a group, while individual older Americans were disproportionately likely to have very high concentrations.

The NHANES data follow the same distribution seen in previous PBDE studies, says Linda Birnbaum, director of the U.S. Environmental Protection Agency’s (EPA) Experimental Toxicology Division. Up to 10% of the population has significantly higher levels in their fat or blood than the rest of the population, and 5% of the population has PBDE levels more than 7 times the median—the outlier population.

“PBDEs are very unusual in that we have seen some people with levels fifty times higher than the median,” Birnbaum explains. The gap between the body burdens of people with the highest levels of exposure and median levels of exposure is much smaller for most other persistent and bioaccumulative chemicals, she explains. For example, about 5% of the population has levels of PCBs and dioxin that are 2 times the median, and 1% has levels that are 3 times the median, she says. The NHANES PBDE data show that the 5% of the population with the highest concentrations of BDE-47 harbor concentrations of the compound that are more than 7 times higher than the geometric mean.

The CDC researchers didn’t report the average concentrations of the top 1% of the NHANES PBDE survey’s participants, but they say the highest value of total PBDEs reported in NHANES was 3,680 ng/g. This is more than 12 times higher than the 95th percentile of total PBDEs in the NHANES data, which was 291 ng/g, and it makes clear that the most highly exposed members of the U.S. population take up much greater quantities of the compounds, says Myrto Petreas, chief of the California Department of Toxic Substances Control’s Environmental Chemistry Branch. Many studies have documented what Birnbaum calls “super highly exposed people” with similarly high—or higher—PBDE concentrations who have no known risk factors for excessive PBDE exposure.

This raises the question of what happens to people who are members of the outlier population but are not aware of it, particularly children, points out Mary Turyk, an epidemiologist at the University of Illinois at Chicago School of Public Health. “People can be outliers with levels of BFRs tens times higher [or more] and nobody knows who, why, where, and when—a sort of Russian roulette,” says Janna Koppe, an emeritus professor of neonatology at the University of Amsterdam. The NHANES data suggest that millions of Americans could be affected by decisions they made as unwitting consumers over the past several decades, Petreas says.

## Exposure Through Dust

Because consumers tend to keep products such as carpeting, couches, and cars for years, if not decades, many homes and offices continue to have potential sources of penta-, octa-, and decaBDE, and their existence is likely to persist for many years, says Tom Webster, associate chairman of the Boston University School of Public Health’s Environmental Health Department. Additionally, Bob Luedeka, executive director of the Polyurethane Foam Association, says a recent report on flame retardants by the Freedonia Group, a market research firm, provides some evidence suggesting pentaBDE is being produced in China and may enter North America in furniture from that country.

Petreas points out that PBDEs can also be emitted from landfills where products are dumped. If products containing PBDEs are incinerated, they can release brominated dioxins and furans. Research published in the 15 August 2007 issue of *Environmental Science & Technology* documents the presence of polybrominated dibenzo-*p*-dioxins and dibenzofurans in air samples from areas in developing nations such as China where electronics are improperly recycled by burning.

Scientists have shown that some PBDEs can volatilize into the air, and research published over the past year by teams led by Manolis Mandalakis at the University of Crete and Stuart Harrad of the University of Birmingham documents high levels of the flame retardants inside automobiles, Internet cafes, and offices. Research published by Webster and colleagues in the 1 July 2007 issue of *Environmental Science & Technology* also found high levels of PBDEs in air from homes, particularly in “personal air” sampled near breathing zones, which Webster says indicates a “personal dust cloud”—the so-called Pigpen effect. In a review published in the January 2008 issue of the *Journal of Exposure Science & Environmental Epidemiology*, Matthew Lorber of the EPA estimated that more than 80% of PBDE exposure is from nonfood sources, mainly exposure to house dust containing PBDEs, from both unintentional ingestion and dermal contact.

In the 1 March 2007 issue of *Environmental Science & Technology*, a group led by Webster published the first research to definitively link PBDE concentrations in house dust with concentrations in the people living in those homes. Scientists believe the distribution of PBDEs in people’s house dust largely mirrors the patterns found in North Americans’ blood, says Heather Stapleton, an environmental toxicologist at Duke University’s Nicholas School of the Environment and Earth Sciences. She says the highest concentration she and collaborator Webster have found is 540,000 ng/g house dust (data unpublished), which is just a bit higher than previously recorded levels in dust from a U.K. home and an airplane presented at the Fourth International Workshop on Brominated Flame Retardants, held 24–27 April 2007 in Amsterdam.

The first evidence that PBDEs in dust are readily available and biologically active was published in the 1 April 2008 issue of *Environmental Science & Technology* by a team led by Janice Huwe of the U.S. Department of Agriculture Biosciences Research Laboratory. The research “demonstrates experimentally and quite conclusively—using real-world house dust—that [PBDEs in dust are] very readily taken up into an animal,” says Birnbaum, who was senior author on the report. “People often think if something is a particulate or bound to dust, it is not going to be getting into our bodies very well. . . . We now know that the PBDEs in the dust being found in our homes and our offices can be taken up by our bodies,” she concludes. Notably, the research refutes the earlier belief that the large size of decaBDE molecules prevents their being taken up and renders them biologically unavailable. It is conceivable that other POPS might behave in the same way if they were found in high levels inside people’s homes, Birnbaum says.

At the April 2007 BFR workshop, Joe Allen, who was then a graduate student in environmental health at the Boston University School of Public Health, presented information about the use of X-ray fluorescence (XRF) to determine which household items contain bromine, a marker for the presence of PBDEs. XRF has been used for years to analyze house paint for the presence of lead, and it is the first technique to appear promising for pinpointing the consumer products that contain bromine.

The handheld XRF analyzers now available are “really the only way to determine if a product may have PBDEs, without actually taking a ‘biopsy’ of the product, which is plainly not feasible for any in-home research study,” says Allen, now a staff scientist at the consultancy Environmental Health & Engineering. He says the work he has done to date, which has been accepted for publication in *Environmental Science & Technology*, suggests that furniture and televisions are the primary sources of the PBDE levels in U.S. house dust.

However, just how PBDEs from consumer goods end up in dust remains what Stapleton calls “one of the big unknowns.” It is also unclear what it is about house dust that makes PBDEs stick to it.

## Routes of Exposure

Since no one intentionally eats dust, how is exposure occurring? Earlier this year, a team led by Stapleton and Webster provided new insight into this riddle by looking at it in a fresh way. Acting on a hunch, they decided to measure whether PBDEs stick to people’s hands. Their research, slated for publication in the 1 May 2008 issue of *Environmental Science & Technology,* documents that people can have “surprisingly” high quantities of PBDEs on their hands, Stapleton says. If people put their hands in their mouths, the PBDEs on their skin may be inadvertently ingested.

Miriam Diamond of the University of Toronto Geography Department says people may be taking up PBDEs on their skin simply by touching household objects. Diamond’s research has demonstrated that minuscule oil droplets broadcast into home air from activities such as cooking can deposit on almost any household surface to produce a thin, sticky film that can trap indoor chemicals. She documented that levels of PBDEs in films on indoor windows were up to 20 times greater than similar films on the outside of those windows in an article published in *Environmental Science & Technology* on 1 February 2004.

Such organic films can build up on nearly all indoor surfaces, including surfaces that have not been previously treated with PBDEs—“any indoor surface where you can get a build-up of dust [is susceptible],” says Tom Harner, a research scientist at Environment Canada. The way Harner envisions it, after PBDEs volatilize into the indoor air—however that happens—they are likely to eventually land on a surface with an organic film. In other words, any surface containing a film is a potential—albeit temporary—sink, he says. When someone touches such a surface, a portion of the organic film and the POPs it contains can be transferred to his or her hands because human hands naturally absorb oils, Harner points out.

Stapleton and Webster say that smoking, nail biting, and eating oily finger foods such as French fries, nuts, and sandwiches with unwashed hands are all routes by which people may unwittingly consume PBDEs. Their research suggests that “hygiene and behavior can have a big impact on [people’s] body burden,” as Stapleton puts it.

## Differential Exposures

Children under the age of 12 weren’t included in the NHANES PBDE study, but the CDC is currently testing pooled samples of blood from children aged 3–11 years to get a better handle on U.S. levels in this age group, says Larry Needham, chief of the Organic Analytical Toxicology Branch of the CDC’s National Center for Environmental Health. The most comprehensive data collected thus far on children’s PBDE levels are from pooled samples from Australia. In research presented at the April 2007 BFR workshop, Leisa-Maree Toms of the University of Queensland and her colleagues reported that the PBDE concentrations in blood taken from children aged 0–4 years were more than 4 times higher than PBDEs in people over age 16. However, pooled data won’t show if some individual children have extraordinarily high level of PBDEs in their blood, Stapleton points out.

Lorber estimates that children take in approximately 7 times more PBDEs each day than adults because they spend so much time putting their hands in their mouths. Stapleton’s new work showing that PBDEs attach themselves to people’s hands suggests the gap may be even larger. In fact, Stapleton says the dose of PBDEs that toddlers can take up by putting their hands in their mouths is approximately equal to the amount infants receive from breastfeeding, which was previously believed to be the greatest source of exposure that a person could receive over the course of his or her lifetime.

On the other end of the age spectrum, the NHANES data show that adults aged 60 and above were more than twice as likely to have PBDE concentrations in their blood that landed them in the high outlier population. “This suggests that older people’s ability to [eliminate] the PBDE compounds is decreased, or that they are getting greater exposure, perhaps because they spend more time indoors,” Stapleton says.

The NHANES data show that men and women tend to have differing proportions of some PBDE compounds in their blood, suggesting the sexes may metabolize PBDEs differently, Stapleton says. “Maybe females are more vulnerable to effects from PBDEs because of that,” she points out, noting that women generally have higher thyroid hormone fluctuations and are more susceptible to disorders and cancer of the thyroid.

Some researchers wonder if there could be a connection between PBDE exposure and thyroid cancer, a disease that disproportionately affects women. The U.S. National Cancer Institute’s 2006 *Annual Report to the Nation on the Status of Cancer* documents that thyroid cancer incidence rates among women have increased since 1981, a time frame that roughly mirrors when PBDEs have been found in the environment.

“Over the past ten years, the incidence of thyroid cancer in women has been increasing faster than any other cancer in either women or men,” says Pamela Horn-Ross, associate director of the Northern California Cancer Center. However, “there has been little research to date on the relationship between PBDE exposures and cancer in humans,” adds Peggy Reynolds, a senior research scientist at the same center.

## Health Effects

Most of our current understanding about how PBDEs affect living organisms comes from animal toxicity studies. “The major PBDE toxicities seen in laboratory tests are toxicities to the liver and thyroid,” says June Dunnick, a toxicologist with the NIEHS. “The National Toxicology Program [NTP] is conducting a cancer study to determine the carcinogenic potential of lower-molecular-weight polybrominated diphenyl ethers [which are associated with the pentaBDE and octaBDE formulations]. Cancer potential is one of the unanswered questions about this set of chemicals, and no one has addressed this issue to date.”

The only carcinogenicity study of a PBDE compound was conducted for decaBDE by the NTP in the 1970s, but the way decaBDE was added to test animals’ food may have led to limited absorption of the chemical, says Birnbaum. Nevertheless, the study revealed that rodents exposed to high doses of decaBDE developed tumors in their livers and thyroid glands.

Experiments conducted over the past decades show that exposure to PBDEs can cause endocrine disruption in amphibians, birds, fish, mice, and rats, including effects on thyroid, ovarian, and androgen functioning, says Birnbaum. When pregnant fish, mice, and rats are exposed to PBDEs, the flame retardants can cause neurodevelopmental effects in their offspring related to cognition, learning, memory, and the ability to respond to novel stimuli. These changes are also seen when infantile animals are exposed to PBDEs. PBDEs have also been linked to alterations in sperm morphology and function as well as to ovarian toxicity in developing animals.

The concentrations associated with these health effects in animal studies are less than 10 times higher than the PBDE concentrations that are now being reported in the most highly exposed segment of North Americans, Birnbaum says. We need more well-designed epidemiologic studies to see if these same effects are occurring in people, says Sjödin.

Meanwhile, recent studies are beginning to sketch out evidence of human health effects. At the April 2007 BFR workshop, Turyk presented the first evidence that PBDEs can alter thyroid hormone levels in humans. Turyk’s data came from a cohort of mostly older men and women who ate Great Lakes fish. She says she is mainly seeing an association between elevated PBDE concentrations and increased T_4_ (thyroxine) in men’s blood.

“We are seeing subtle changes in some of the thyroid hormone levels that appear to be related to PBDE body burden after controlling for possible confounding by age, body mass index, lipids, and medications,” Turyk explains. She stresses that she excluded people with existing thyroid disease and those taking hormones, and that the link is independent of the cohort population’s levels of PCBs and DDE (the chief metabolite of DDT). PBDE exposure has been inversely correlated with T_4_ levels in animal studies, Birnbaum points out.

Similarly, workers exposed to PBDEs by working in or living near a recycling center in China were more likely to have elevated levels of thyroid-stimulating hormone (TSH) compared with other citizens, according to a study led by Jing Yuan of Huazhong University of Science and Technology published 15 March 2008 in *Environmental Science & Technology*. Elevations of TSH are indicative of stress on the thyroid system, Birnbaum says.

A study published in the March 2008 issue of *EHP* by Maria Athanasiadou of Stockholm University and colleagues documents for the first time that hydroxylated PBDE metabolites can bioaccumulate in human blood serum. Hydroxylated metabolites of PBDEs were shown in the July 2000 issue of *Toxicological Sciences* to compete with thyroid hormones in blood to access transport proteins, although it is not clear if this also happens in humans, says Timo Hamers, an environmental toxicologist at Amsterdam’s Institute for Environmental Studies. Research presented at the April 2007 BFR workshop by Rocío Fernández Cantón of Utrecht University also documents that hydroxylated PBDEs are antiandrogenic.

Even small perturbations of thyroid hormones can have a negative influence on early fetal brain development, according to a study published in September 2003 in *Clinical Endocrinology* by Victor Pop of Tilburg University. Pop’s findings document that women who at 12 weeks gestation had low T_4_ concentrations—though still within what is considered to be the normal range—bore children who demonstrated impaired mental and motor functioning at 1 to 2 years of age. (This study examined thyroid perturbation in general, not in relation to PBDE exposure.)

Researchers have known for several years that babies can have detectable levels of PBDEs in their blood. In a study published in the July 2003 issue of *EHP*, researchers led by Anita Mazdai of the Indiana University School of Medicine reported that the concentrations of PBDEs in umbilical cord blood from a group of 12 infants ranged from 14 to 460 ng/g lipid and correlated well with the concentrations in their mothers. More recently, a team led by Lynn Goldman of the Johns Hopkins Bloomberg School of Public Health collected cord blood from a cohort of 297 Baltimore babies who have concentrations of PBDEs similar to those of the Indiana babies, including a few samples with levels much higher than the median for this group. Goldman is investigating what may be an association between PBDE concentrations and thyroid hormones in the Baltimore cohort.

Elevated PBDE concentrations in mothers’ milk were correlated with cryptorchidism (undescended testes) in their children in a study published in the October 2007 issue of *EHP* by Katharina Maria Main of Rigshospitalet University. At 4.16 ng/g, PBDE concentrations associated with cryptorchidism in that study were much lower than those reported in the Mazdai study. Turyk points out that the PBDE levels associated with the cryptorchidism reported in the Main study “are seven times lower on average than those in our adult population.”

Koppe says, “it is certainly possible that there is a link between PBDE exposure in the fetus and cryptorchidism.” However, in a letter published in the May 2008 issue of *EHP*, she notes that 4 of 33 Finnish boys and 1 of 28 Danish boys with cryptorchidism had mothers with diabetes, a known major cause of congenital malformations. Main counters that although the original analysis did not correct for diabetes, a reanalysis of the data omitting diabetic mothers still returned a significant association between PBDEs in breast milk and cryptorchidism [for the complete exchange, see Correspondence, p. A195 this issue].

## More BFR Research in the Future

Elaine Ron, a senior investigator and epidemiologist at the National Cancer Institute, says her institute is trying to find a way to explore whether there could be a link between PBDEs and papillary thyroid cancer, the form that has been increasing most rapidly, in any of the cohorts currently being studied for thyroid cancer risk factors. “Thyroid cancer is pretty much of an enigma. We know that radiation can increase risk quite dramatically depending upon the age of exposure, but other risk factors are not very clear,” Ron says.

A number of studies are gearing up to further investigate how maternal PBDE exposures affect thyroid function in children. For example, the Chemicals, Health and Pregnancy (CHirP) study, being led by a team of researchers from the University of British Columbia has just finished recruiting 150 pregnant women and will assess levels of both PBDEs and thyroid hormones, says Glenys Webster, the study’s director. “We now know that small changes in thyroid hormone levels—especially during early pregnancy—may affect neurological development in children. Since everyone is exposed to PBDEs, even small effects on thyroid hormones may therefore be of concern for public health,” she says.

Koppe notes that if mothers with low T_4_ levels are identified early on in their pregnancies, the reduction can be compensated for to minimize its effects. Webster adds that if her study ferrets out developmental effects, her next step would be to try to find funding for a case–control study to examine children for possible links to attention deficit/hyperactivity disorder, autism, and learning disabilities.

Research such as the CHirP study is important because the thyroid perturbations documented to date in people “may not be clinically significant in an individual, but could contribute to overall disease burden in the population,” Turyk says.

Besides PBDEs, the CHirP study will also measure participants’ concentrations of PCBs, organochlorine pesticides, and polyfluorinated compounds including perfluorooctane sulfonate (PFOS) and perfluorooctanoic acid (PFOA). Stapleton says she is also planning to participate in a similar study being funded by the CDC. In 2006, Stapleton helped the National Institute of Standards and Technology produce an official Standard Reference Material that documents the presence in house dust of 33 polycyclic aromatic hydrocarbons, 30 PCBs, and 4 chlorinated pesticides in addition to 15 PBDE compounds.

Over the past year, a number of researchers reported finding at least five other bromine-containing flame retardants in household dust in the same skewed patterns that have been documented for PBDEs. Stapleton says she, too, detected flame retardants besides PBDEs on people’s hands. The implications are significant, she points out, “once you start to think about all the chemicals in dust that [people] are exposed to.”

## Figures and Tables

**Figure:**
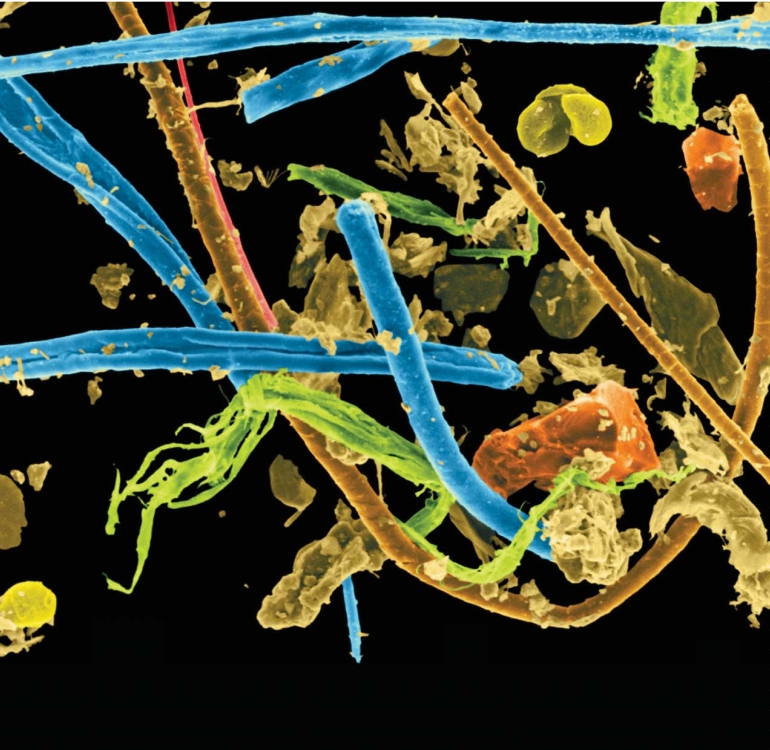
Although house dust is known to be a predominant source of exposure to PBDEs, it’s not yet clear which part of the dust these chemicals bind to. The dust pictured above contains pet hair (rust brown), pollen (yellow), plant fibers (green), dead skin cells (light to medium brown), dirt and minerals (orange), textile fibers (blue), and spider silk (pink).

**Figure:**
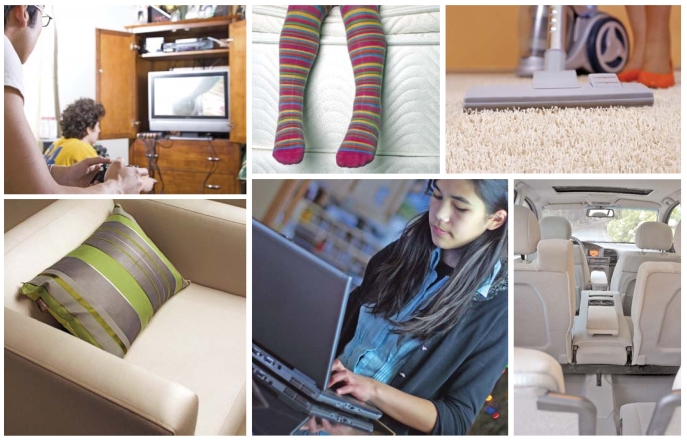
PBDEs are used in a number of consumer goods, including video and computer equipment, cell phones, mattresses, upholstered furniture, carpet padding, and automobile electronics and seats. Virtually all samples tested for PBDEs in the National Health and Nutrition Examination Survey contained BDE-47.

**Figure:**
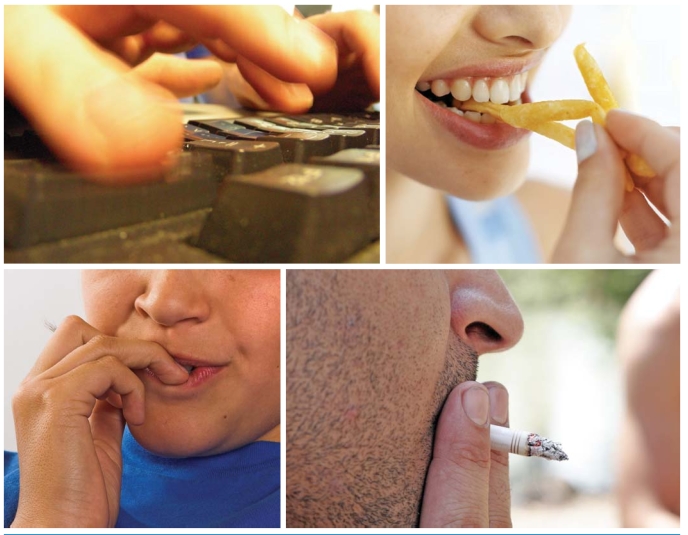
Hand-to-mouth exposure is thought to account for much of people’s intake of PBDEs. Although this may help explain why some of the highest concentrations of PBDEs have been found in children’s blood, hand-to-mouth exposure isn’t just for toddlers—adults may unwittingly consume the chemicals as they smoke, eat, or bite their nails.

